# GluR2 endocytosis-dependent protein degradation in the amygdala mediates memory updating

**DOI:** 10.1038/s41598-019-41526-1

**Published:** 2019-03-26

**Authors:** Nicole C. Ferrara, Timothy J. Jarome, Patrick K. Cullen, Sabrina A. Orsi, Janine L. Kwapis, Sydney Trask, Shane E. Pullins, Fred J. Helmstetter

**Affiliations:** 1 Department of Psychology, University of Wisconsin-Milwaukee, Milwaukee, WI USA; 2 Department of Animal and Poultry Sciences, Virginia Polytechnic Institute and State University, Blacksburg, VA USA; 3 School of Neuroscience, Virginia Polytechnic Institute and State University, Blacksburg, VA USA

## Abstract

Associations learned during Pavlovian fear conditioning are rapidly acquired and long lasting, providing an ideal model for studying long-term memory formation, storage, and retrieval. During retrieval, these memories can “destabilize” and become labile, allowing a transient “reconsolidation” window during which the memory can be updated, suggesting that reconsolidation could be an attractive target for the modification of memories related to past traumatic experiences. This memory destabilization process is regulated by protein degradation and GluR2-endocytosis in the amygdala. However, it is currently unknown if retrieval-dependent GluR2-endocytosis in the amygdala is critical for incorporation of new information into the memory trace. We examined whether the addition of new information during memory retrieval required GluR2-endocytosis to modify the original memory. The presentation of two foot shocks of weaker intensity during retrieval resulted in GluR2 endocytosis-dependent increase in fear responding on a later test, suggesting modification of the original memory. This increase in fear expression was associated with increased protein degradation and zif268 expression in the same population of cells in the amygdala, indicating increased destabilization processes and cellular activity, and both were lost following blockade of GluR2-endocytosis. These data suggest that the endocytosis of GluR2-containing AMPA receptors in the amygdala regulates retrieval-induced strengthening of memories for traumatic events by modulating cellular destabilization and activity.

## Introduction

Memory formation is a dynamic process that involves several phases. Following the initial learning event, memories undergo a time-dependent process at the molecular level known as consolidation^1,2^. Once consolidated, memories are considered to be stable and no longer susceptible to disruption. However, following retrieval, previously formed memories “destabilize” and require new protein synthesis to “restabilize”, a process referred to as reconsolidation^3^ which allows for the modification of the original memory. Consistent with this, numerous studies have shown that reconsolidation can be used to strengthen, weaken or change the specific content of a memory (e.g.^4,5^). For these reasons, the reconsolidation process has become an attractive target for therapeutic strategies designed to treat maladaptive memories associated with many anxiety disorders such as post-traumatic stress disorder^6–8^. Some recent evidence has shown that pharmacological blockade of reconsolidation in humans can be used to treat phobias, supporting its therapeutic efficacy^9^. However, much remains unknown about the molecular mechanisms that initiate the reconsolidation process and whether they are the same as those that are involved in the “modification” of memories for traumatic events.

Pavlovian conditioning rapidly produces a fear memory that is robust and long-lasting. During training, a neutral conditional stimulus (CS) is paired with an aversive unconditional stimulus (UCS). As a result of this association, the CS acquires aversive value and forms a fear memory that can be measured by the ability of the CS to elicit a conditioned response in the absence of the UCS. Pavlovian fear memories require the amygdala for their acquisition and long-term storage, and pharmacological manipulations in the amygdala can have dramatic effects on the consolidation and reconsolidation of fear memories^3,10–12^. For example, numerous studies have demonstrated that fear memory formation requires *de novo* protein synthesis and protein degradation and is characterized by increased surface expression of AMPA receptors at amygdala synapses^13–15^. AMPA receptors can be classified based on calcium permeability. Calcium-permeable AMPA receptors (CP-AMPARs), or those that lack the GluR2 subunit, are thought to be less stable and are trafficked into the synapse following learning^13,16^. Since these receptors allow calcium influx and thus promote plasticity, the replacement of CP-AMPARs with CI-AMPARs has been proposed as a mechanism by which the memory stabilizes and becomes resistant to disruption.

Retrieval of a stored memory induces a period of AMPAR exchange at synapses in the amygdala, where CI-AMPARs are transiently replaced with CP-AMPARs^13^. This exchange of AMPARs is critical for the destabilization of the memory trace, and is thought to be regulated by activity from the ubiquitin proteasome system^14,17,18^. Specifically, stable synaptic scaffolds anchoring AMPA receptors can undergo proteasome-dependent degradation to allow for internalization of AMPARs^19,20^, suggesting that proteasome activity may be critical for CI-AMPAR-mediated synaptic destabilization during memory reconsolidation. However, it is unknown if proteasome activity is involved in the exchange of AMPAR complexes following retrieval. Furthermore, a majority of the existing work characterizing a necessity for specific cellular mechanisms in memory destabilization during reconsolidation relies on the rescue of the memory from an amnesic agent^14,17,21^, so it is unclear if AMPAR-mediated destabilization is critical for memory modification when new cues are presented during a retrieval session.

In the current experiments, we studied AMPAR exchange in regulating reconsolidation-dependent memory destabilization and modification when new information about the UCS is included during the retrieval session. We show that low intensity UCS presentations during the retrieval session strengthen fear expression and that this is likely regulated by reconsolidation mechanisms since it is dependent on amygdala protein degradation and GluR2 endocytosis. Furthermore, increased fear responding in response to the additional UCS presentations increased lateral amygdala cellular activity and ubiquitination in similar cell populations, which was reversed by pharmacological blockade of GluR2 endocytosis. Collectively, our data suggest that CI-AMPAR endocytosis regulates reconsolidation-dependent modification of a fear memory in the amygdala.

## Materials and Methods

### Subjects

Male Long-Evans rats were obtained from Envigo (Frederick, MA and Madison, WI) and weighed between 300 and 350 g at the time of arrival. Animals were individually housed with free access to water and food. All procedures were approved by the University of Wisconsin-Milwaukee and the Virginia Polytechnic Institute and State University Institutional Animal Care and Use Committees and were conducted according to the ethical guidelines of the National Institutes of Health.

### Surgery

Immediately before surgery, rats were anesthetized with 4% isoflurane and oxygen, and after induction, isoflurane levels were maintained at 2–2.5% throughout the surgery. Using stereotaxic coordinates referenced to bregma, animals were implanted with bilateral cannulae targeting the amygdala (−3.0 mm posterior, +/−5.0 mm lateral, −7.2 mm ventral). Each cannula was secured to the skull with a stainless steel screw and surrounded by acrylic cement. A dummy cannula was screwed into the cannula guide to prevent occlusion.

### Infusion Procedure

Rats received bilateral microinjections of clasto lactacystin β-lactone (32 ng/μl, Sigma), GluR23Y (30pmol/μl, AnaSpec), TAT-GluR2_3A_ fusion peptide (30pmol/μl, AnaSpec), or ACSF at a rate of 0.25 μl/min and at a volume of 0.5 μl/hemisphere 60 minutes before a brief retrieval session^14,22^. Rats were returned to their home cages after injections.

### Apparatus

**Figure 1:**
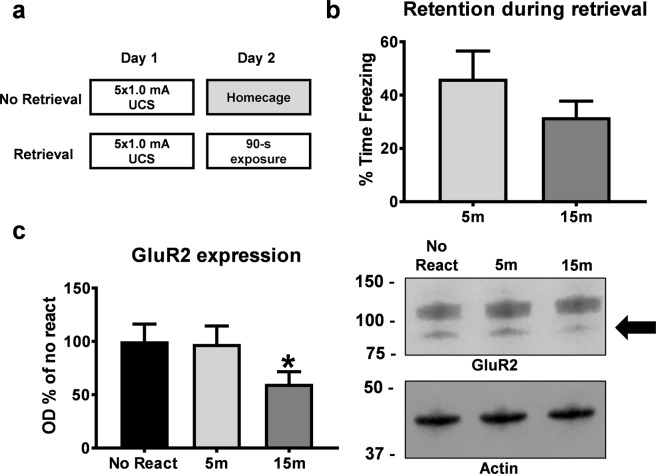
Contextual fear memory retrieval is characterized by decreases in GluR2 surface expression in the amygdala. (**a**) Experimental Design. Rats were trained to contextual fear conditioning and re-exposed to the training context for 90 sec the following day. Animals were sacrificed between 5- and 15-min after context fear memory retrieval and the amygdala collected (n = 8 per group). Groups are normalized to the no reactivation control group. (**b**) There were no differences between groups during retrieval. (**c**) Retrieval of a context fear memory decreased GluR2 expression 15 min, but not 5 min, after retrieval. Representative GluR2 and Actin western blot bands are next to the graph. Full-length membranes are presented in Supplemental Fig. 1. *P < 0.05 from No React.

**Figure 2:**
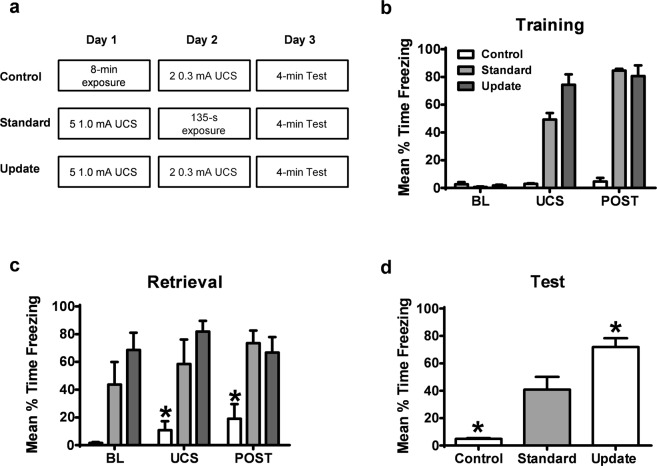
Presentation of low intensity shocks during retrieval results in memory strengthening selectively in groups that were contextually fear conditioned. (**a**) Experimental design. Standard retrieval and Update groups were contextually fear conditioned and control animals were exposed to the context for equivalent duration on Day 1. The following day, Update and Control groups were subject to 2 lower intensity footshocks while the Standard retrieval group was placed into the training context for an equivalent duration. Averages of freezing during the UCS periods reflect behavioral performance during and between all UCS presentations. On the third day, all groups were tested for their retention to the training context. (**b**) Animals in Standard and Update groups, but not the control animals, displayed increased fear to the context through the training session. (**c**) During retrieval, animals that received two lower intensity footshocks without conditioning had poorer memory for the training context in comparison to animals previously conditioned. (**d**) Animals that were not conditioned on Day 1 but received low intensity footshocks on Day 2 show less fear to the context than the Standard retrieval group. However, animals that were fear conditioned and received low intensity footshocks during retrieval show significantly higher freezing when compared to the standard retrieval group, indicating strengthening of the memory.

**Figure 3:**
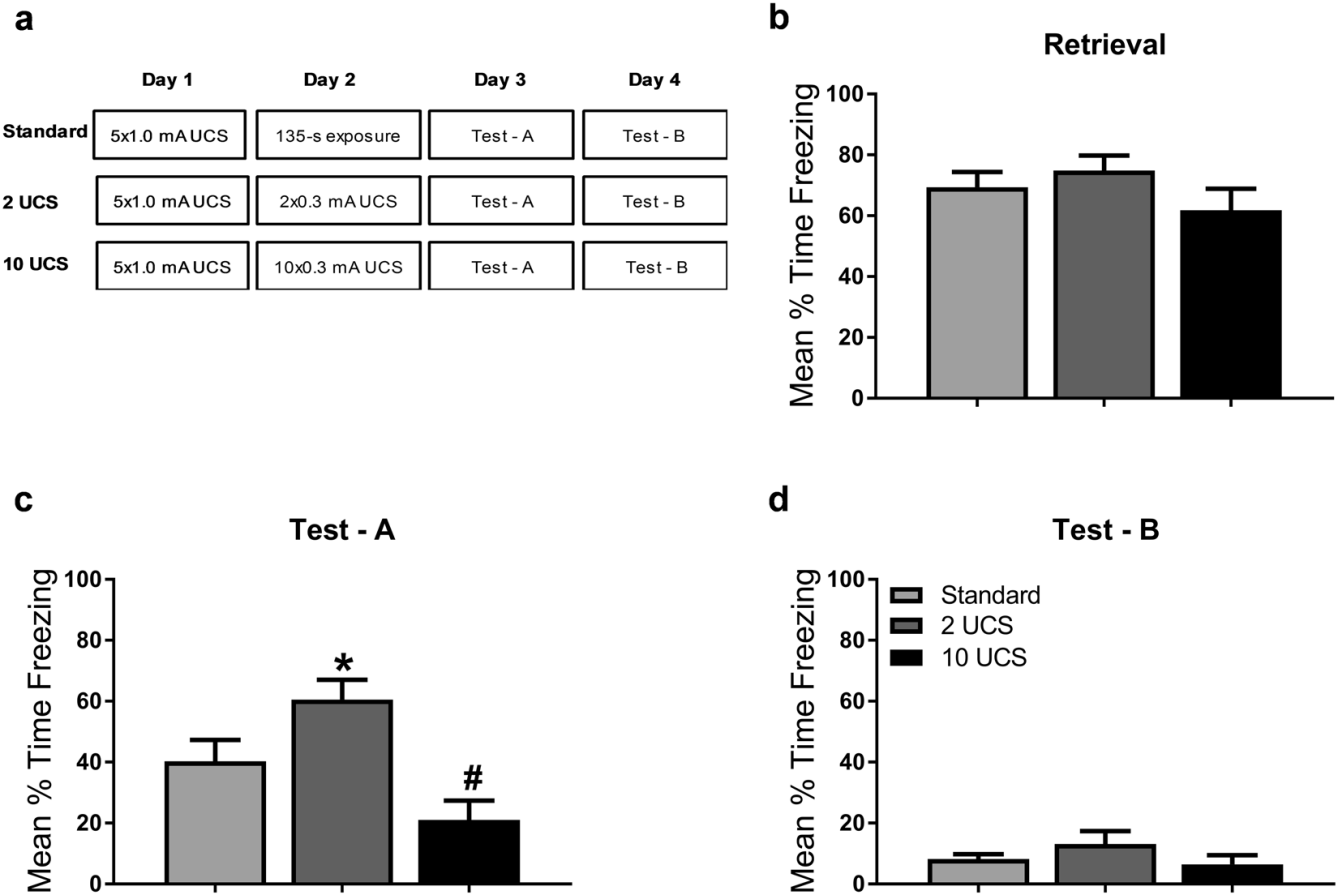
Increases in fear responding following two lower intensity footshocks is not due to stress-enhanced fear learning. (**a**) Experimental design. Standard retrieval, 2UCS and 10UCS update groups were contextually fear conditioned on Day 1. The following day, the update groups received 2 (2UCS) or 10 (10UCS) lower intensity footshocks while the Standard retrieval group was placed into the training context for an equivalent duration. On the third day, all groups were tested for their retention to the training context. Then, on day 4, animals were placed in a novel context to assess fear generalization. (**b**) There were no differences in memory retention between groups during retrieval. (**c**) During test, animals that received 2 lower intensity footshocks show significantly higher freezing when compared to the standard retrieval group, indicating strengthening of the memory, while animals that received 10 lower intensity footshocks had decreased fear responding, indicating memory weakening. (**d**) None of the groups showed fear to a novel context, indicating a lack of fear generalization. *P < 0.05 from Standard retrieval. ^#^*P* = 0.053 from Standard retrieval.

**Figure 4:**
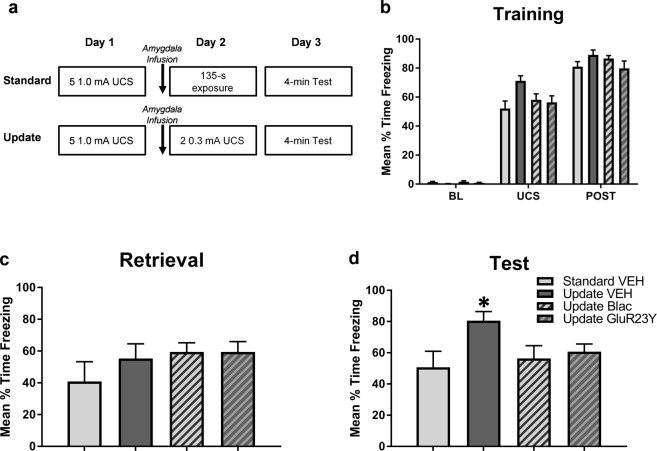
Retrieval-dependent memory strengthening is regulated by protein degradation and GluR2 endocytosis in the amygdala. (**a**) All groups were contextually fear conditioned and 24-hours later received infusions into the amygdala 1-hr prior to a brief retrieval (standard) or update session. Standard retrieval groups received infusions of vehicle (TAT-GluR2_3A_ fusion peptide or 2% DMSO in ACSF), and Update groups received infusions of vehicle, GluR23Y (GluR2 endocytosis blocker) or β-lac (proteasome inhibitor). The next day, animals were tested for fear memory retention. (**b**) All groups show similar fear expression during the post-shock period of the training session, suggesting they all acquired the task normally. (**c**) There were no differences between groups during the retrieval. (**d**) Animals that received vehicle infusions prior to the update show elevated freezing in comparison to the Standard retrieval group, which was prevented in animals that received β-lac or GluR23Y infusions. *P < 0.05 from Standard retrieval.

**Figure 5:**
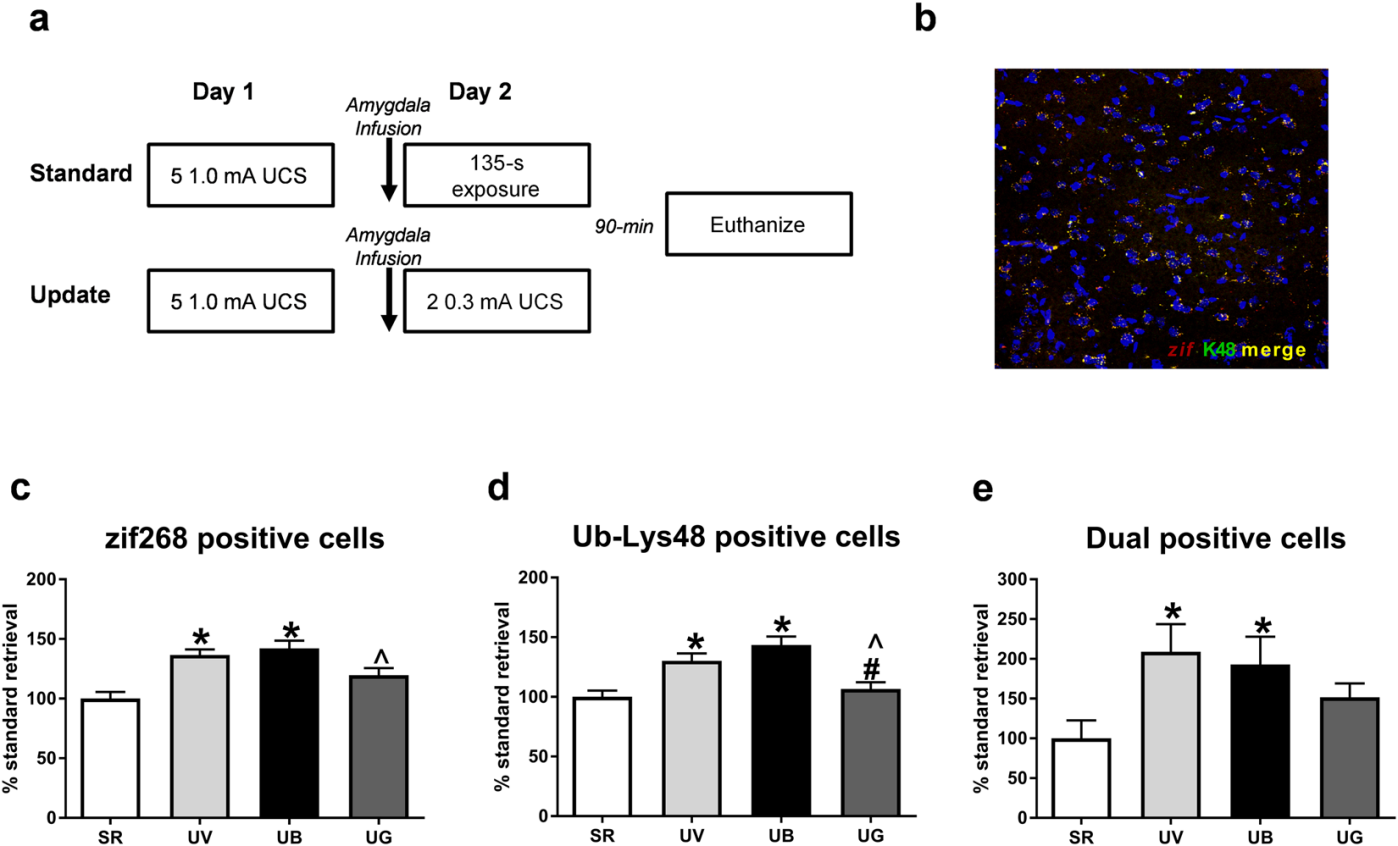
Memory updating increases zif268 and Ub-Lys48 expression in similar cell populations in the amygdala and is regulated by endocytosis of GluR2. (**a**) All groups were contextually fear conditioned and 24-hours later received infusions into the amygdala 1-hr prior to a brief retrieval (standard) or update session. Standard retrieval groups received infusions of vehicle (TAT-GluR2_3A_ fusion peptide or 2% DMSO in ACSF), and Update groups received infusions of vehicle, GluR23Y (GluR2 endocytosis blocker), or β-lac (proteasome inhibitor). Following update or retrieval, animals were euthanized 90 minutes later and tissue was processed for immunofluorescence. (**b**) Example immunofluorescence image used for cell counting in the lateral amygdala. LUT parameters were modified only for this image to minimize background (**c**) There were more zif268 expressing cells in the amygdala of animals receiving the Update procedure, which was reduced by blocking GluR2 endocytosis but not proteasome activity. (**d**) There were more Ub-Lys48 expressing cells in the amygdala of animals receiving the Update procedure, which was reduced by blocking GluR2 endocytosis but not proteasome activity. (**e**) The Updating procedure increased the amount of zif268 and Ub-Lys48 dual expressing cells in the amygdala which was reduced by the GluR23Y peptide but not the proteasome inhibitor. *P < 0.05 from standard retrieval. ^#^*P* < 0.05 from Update vehicle. ^*P* < 0.05 from Update Blac.

For Fig. 1, animals were trained with contextual fear conditioning in a Habitest behavioral apparatus developed by Colbourn Instruments (Holliston, MA). This chamber consisted of a 10 × 12 × 12 (LxWxH) steel test cage with front and back Plexiglas walls and a grid shock floor above a plastic drop pan. The right wall of the chamber consisted of a house light in the top back corner, which remained on during the behavioral procedures, and all remaining slots of both walls were filled with blank metal panels. A USB camera was mounted on a steel panel outside the back Plexiglas wall of the chamber, angled at ~45 degrees. The entire chamber was housed in an isolation cubicle with an acoustic liner and a house fan, which remained active during all behavioral procedures. Shock was delivered through the grid floor via a Precision Animal Shocker under the control of FreezeFrame 4 software. For Figs 2–5, animals underwent contextual fear conditioning in a set of four Plexiglas and stainless steel chambers within sound-attenuating boxes. The floor consisted of 18 stainless steel bars connected to a shock generator (Coulbourn Instruments). Each chamber contained ventilation fans to provide a constant background noise (approximately 60dB). Between rats, the chambers were cleaned with 5% ammonium hydroxide solution.

### Behavioral Procedures

For contextual fear conditioning, animals were placed into the training apparatus where they received 5 unsignaled foot shocks (1 sec, 1.0 mA) after a 2 min baseline period^14,23^. In cases where animals underwent surgical procedures, animals recovered for 4–7 days and were then adapted to transport handling procedures for 3 days before conditioning. The transport handling procedures included a gentle restraint during the sound of the infusion pump. Following completion of this habituation phase, animals underwent two different experimental procedures. In Experiment 1, rats were trained with contextual fear conditioning as described above and the following day exposed to the training context for 90 sec in the absence of shock to reactivate the memory. A second group of animals was trained but did not receive context exposure on Day 2 and served as a no retrieval (No React) control group. For Experiments 2–5, animals were either trained with contextual fear conditioning as described above or exposed to Context A for 8 minutes without receiving any shock presentations (Control). On day 2, animals that were trained with contextual fear conditioning on Day 1 were placed back into the training context for a retrieval or update procedure. Standard groups received a retrieval condition where animals were exposed to the chamber for 135 sec in the absence of footshock while the Update group received two or ten unsignaled foot shocks at a reduced intensity (0.3 mA) after a 60 sec baseline. The Control group was also given the two weaker intensity foot shocks on Day 2. Animals subjected to the lower intensity shock procedure were removed from the context 15 sec after the final foot shock. All groups had equivalent exposure times to Context A throughout the experiment. On Day 3, all groups were placed back into the context in the absence of shock for a 4 minute test to measure the strength of context fear memory.

### Tissue Collection and Crude Synaptosomal Membrane Preparation

Animals were deeply anesthetized with isoflurane, decapitated and brains removed and stored at −80 °C until dissected. Crude synaptosomal membrane fractions were obtained as previously described^14,24^. Amygdala samples were homogenized in TEVP buffer with 320 mM sucrose, and were then centrifuged at 1000 × g for 10 minutes. The supernatant was removed and centrifuged at 10,000 × g for 10 minutes, and the remaining pellet was denatured in lysis buffer. Supernatant was collected and measured using a Bradford protein Dc assay kit (BioRad, Hercules, CA).

### Antibodies

Western blot antibodies included GluR2 (1:1000, NeuroMab #75-002) and Actin (Cell Signaling, 1:1000 #4697) as a loading control. Immunofluorescence antibodies included zif268 (Cell Signaling, 1:500) and Anti-Ubiquitin linkage-specific Ub-Lys48 (Alexa Fluor® 488; Abcam, 1:100).

### Western blotting

Samples (10 μg) were loaded on 7% Acrylamide gels, ran through SDS-PAGE and transferred using a Turbo Transfer System (Biorad). Membranes were incubated in a 50:50 blocking buffer (50% Licor TBS blocking buffer and 50% TBS + 0.1% Tween-20) for 1 h at room temperature, followed by an overnight incubation in primary antibody in 50:50 blocking buffer at 4 °C. Membranes were then washed 3 times for 10 min with TBS + 0.1% Tween-20 (TBSt) and incubated in secondary antibody (1:20,000; goat anti-rabbit 700CW or goat anti-mouse IgG1 800CW) in 50:50 blocking buffer for 45 min. After two 10 min washes in TBSt, the membranes were washed in TBS and imaged using the LI-COR Odyssey Fc. Visualized proteins were analyzed using Image Studio Ver 5.2.

### Immunofluorescence

Animals were deeply anesthetized with isoflurane 90 min following retrieval or update. Brains were immediately removed and stored at −80 °C until sliced. Brains were sliced in 40 micron sections and were mounted onto charged slides. Slides were rehydrated in wash buffer (PBS + 0.05% Tween-20) and permeabilized (PBS + 0.3% Triton X) for 15 min, and incubated in blocking solution (PBS + 0.7% NGS). Slides were then incubated in zif268 antibody solution (PBS + 0.3% Triton X + 5% NGS) overnight at 4 °C. The next day, slides were incubated in secondary antibody solution for 2 hours and rinsed with wash buffer. Ub-Lys48 conjugated antibody solution was then applied, and slides incubated overnight at 4 °C. The following day, slides were rinsed, a DAPI counterstain was applied, and slides were cover slipped.

### Immunofluorescence microscopy and quantification

Specific anatomical locations were chosen based on a brain atlas^25^. Amygdala images were captured on an Olympus Fluoview FV1200 confocal microscope using a 40x oil immersion lens. Serial *z*-stack images covered a depth of 4.55μm through five consecutive sections (0.91μm per section) and were acquired using Fluoview software (Olympus). The LUT was linear and covered the full range of data for all quantified images (0–4095). For the displayed image in Fig. 4, LUT parameters were modified to remove background and for signal clarity (blue: 355–4095, red: 446–3238, green: 736–2940). Three amygdala sections were captured and analyzed bilaterally and were averaged for each rat (4–6 sections matched along the anterior-posterior axis for each rat in the final analysis).

Images were exported as 12-bit TIFF files and particles were quantified using ImageJ software (NIH, Bethesda, MD, USA). Images were quantified by converting them to 32-bit, difference of Gaussian filtering (sigmas of 2 and 1.5), thresholding with the triangle method, and then counting particles greater than 4 pixels in diameter within the ROI. Merged particles were counted based on colocalization of a stacked RGB image in ImageJ. Colocalized points were considered overlapping particles in both red and green channels if intensities were higher than predetermined thresholds based on intensity of red and green merged sections that excluded background and any particles that were exclusively red or green. All particle counts were averaged, bilaterally, across animals in each condition and normalized to standard retrieval control slices using the “Analyze Particles” plugin in ImageJ.

### Conditioned fear responses

Freezing behavior was measured for each rat during each context exposure and was defined as the cessation of all movement excluding respiration and was automatically scored in real-time with FreezeFrame 4 (Fig. 1) or FreezeScan 1.0 detection software (Clever Sys, Inc., Reston, VA) calibrated to a trained human observer.

### Statistical Analyses

All statistical analyses and graphing were conducted in GraphPad. Behavioral and western blot statistical outliers were defined as two standard deviations above or below the group mean. The data are presented as group averages with standard error of the mean (SEM). Experimental results were analyzed using a one-way Analysis of Variance (ANOVA) with the exception of when Levene’s test was violated and the corrected Welch statistic was calculated in SPSS and used in place of the uncorrected one-way ANOVA. Independent samples one-tailed t-tests were used when comparing western blot samples, and Dunnet *post hoc* tests were used for behavior and immunofluorescence when appropriate to compare the standard retrieval condition to all other groups.

## Results

### Contextual fear memory retrieval is characterized by decreases in GluR2 surface expression in the amygdala

We first tested whether the retrieval of a contextual fear memory changes GluR2 subunit protein expression in the amygdala. Animals were trained with context fear conditioning and given a brief retrieval 24 hours later (Fig. 1a). There were no differences between groups during the retrieval session (t_(14)_ = 1.205, *P* > 0.05; Fig. 1b). We then collected amygdala crude synaptsomal membrane fractions 5 or 15-mins later and examined expression of GluR2 subunits using western blotting. We chose these time points based on previously reported changes in AMPA:NMDA ratios during memory reconsolidation in the amygdala^13^. We found that GluR2 surface expression decreased 15- (*t*_(13)_ = 1.951, *P* = 0.0365), but not 5-min (*t*_(14)_ = 0.09695, *P* > 0.05), after retrieval relative to no retrieval controls (Fig. 1c). This suggests that contextual fear memory retrieval is characterized by decreases in the expression of GluR2 containing AMPARs, which supports previous studies indicating changes in GluR2 protein expression following retrieval^13,26^. Cropped representative bands are displayed in Fig. 1 and full-length blots are presented in Supplemental Fig. 1.

### Low intensity shocks during a single retrieval session result in memory strengthening in previously trained groups

Having established that retrieval of a contextual fear memory changes the expression of GluR2 subunits in the amygdala, we next wanted to test if GluR2 trafficking in the amygdala was critical for memory updating following retrieval. To address this, we first tested if we could modify a strongly learned contextual fear memory. Incorporation of new information during retrieval can be characterized by changes in behavioral expression of fear^22,27^. Thus, we examined whether the strength of the memory could be changed following retrieval by the incorporation of new information regarding the shock itself (Fig. 2a). We trained animals with contextual fear conditioning as in the previous experiment and on the second day gave them a brief exposure to the training context. During the re-exposure, animals either did not receive the shock (Standard) or were presented with a shock presentation of considerably lower intensity than they had received during training (Update). Lower shock intensities in the Update condition were used to avoid resistance to pharmacological intervention by engaging reconsolidation boundary conditions through asymptotic learning of fear with multiple strong UCS presentations^28,29^. A separate group of animals was exposed to the context on Day 1 with no shock presentations, followed by two low intensity shock presentations on Day 2 (Control).

On the first day, both Standard and Update groups showed equal levels of freezing during the post-shock training period (*F*_(4,33)_ = 31.69, *P* > 0.05), an indicator of short-term memory for the task, while the Control group showed significantly lower freezing than groups undergoing conditioning (*P* < 0.01; Fig. 2b). Similarly, the following day, there were no significant differences in freezing responses between Standard and Update groups during the retrieval session, despite the additional low intensity shocks presented to the Update group (*F*_(4,33)_ = 0.642, *P* > 0.05). Additionally, the Control group maintained low freezing levels in response to the presentation of 2 low intensity foot shocks on Day 2 compared to the Standard and Update groups (*P* < 0.05; Fig. 2c). The Control group showed significantly less freezing than the Standard group on the third day (*F*_(2,11)_ = 21.6, *P* < 0.01), while the Update group showed significantly elevated freezing in comparison to the Standard group (*P* < 0.05; Fig. 2d). Collectively, these results indicate that exposure to low intensity foot shocks strengthened the fear response measured on later tests, suggesting that this fear memory persists even in the face of new information about the original CS-UCS relationship.

Previous stress enhanced fear learning work shows that strong conditioning can result in potentiated fear responding that is due to shock sensitization^30^. However, UCS devaluation work suggests that decreasing the shock intensity should reduce fear responding. We wanted to determine whether the increase in fear responding at test as a result of two lower intensity foot shocks was due to 1) overall amount of shock exposure and engagement of non-associative mechanisms (e.g. sensitization) or 2) associative mechanisms that require additional UCS exposures (e.g. devaluation). Here, we included an additional group that received 10 lower intensity UCS presentations on the second day (Fig. 3a**)**. Persistent increases in fear responding after mulitple low intensity UCS presentations would suggest these results are similar to what is seen in stress enhanced fear learning, while decreases in fear responding would suggest these results are consistent with modification of the memory formed at training. There were no significant differences during the retrieval session (*F*_(2,19)_ = 1.254, *P* > 0.3, Fig. 3b) when groups were re-exposed to the context in the absence of shock or when multiple lower intensity shocks were presented. During the test in the conditioning context, we replicated the significant increases in fear responding as a result of two low intensity foot shocks (*F*_(2,19)_ = 8.348, *P* < 0.05, Fig. 3c) and a strong trend for a decrease in freezing when groups were exposed to ten low intensity foot shocks compared to the Standard group (*P* = 0.053). Several presentations of strong shock could result in generalization of fear^31^, so we included an additional test in a distinct context to test for fear generalization. Exposure to a novel context after conditioning and update procedures did not result in generalization of fear between groups with different shock exposures (*F*_(2,19)_ = 1.232, *P* > 0.314, Fig. 3d). These results indicate that the increases in fear in conditioned groups as a result of two lower intensity foot shocks is not engaging similar mechanisms to stress enhanced fear learning and does not result in significant generalization.

### Retrieval-dependent memory strengthening requires protein degradation and GluR2 endocytosis

The strengthening and weakening of fear response can share similar mechanisms during reconsolidation-dependent memory modification^4,13,26^. For the following experiments, we focused on the two UCS Update condition to test whether the additional shock presentations during retrieval engage GluR2-dependent reconsolidation mechanisms to strengthen the fear memory. We repeated the Standard and two UCS Update procedure from the previous experiments in combination with pharmacological manipulations of either GluR2 endocytosis or proteasome activity in the amygdala. In this experiment, the Update group received the GluR2 endocytosis inhibitor, GluR23Y, the proteasome inhibitor, β-lac, or vehicle into the amygdala immediately prior to context re-exposure (Fig. 4a). Groups show similar freezing responses during contextual fear conditioning (*F*_(6,76)_ = 2.138, *P* > 0.05; Fig. 4b). Drug infusions into the amygdala did not impact fear responding during the retrieval/update between groups (*F*_(3,38)_ = 3.707, *P* > 0.05; Fig. 4c). However, at test, the vehicle Update group showed significantly more freezing to the context than the Standard group (*F*_(3,37)_ = 4.005, *P* < 0.05), which was prevented with infusions of β-lac or GluR23Y in the amygdala (Fig. 4d). These results suggest that the potentiated freezing response elicited by additional shocks during retrieval are engaging reconsolidation-related molecular mechanisms. Specifically, amygdala GluR2 endocytosis and protein degradation are necessary for the incorporation of new information during the retrieval session to update the original fear memory.

### Memory updating increases zif268 and Ub-Lys48 expression in similar cell populations and is regulated by endocytosis of GluR2s

Our previous experiment revealed that GluR2 endocytosis and protein degradation were critical for the retrieval-dependent strengthening of a contextual fear memory. However, it is unclear if the memory strengthening effect is occurring in the same cells in which the original memory was undergoing destabilization. To test this, in our final experiment we examined whether cellular markers of memory destabilization overlapped with markers of cellular activity in the amygdala following our memory updating procedure. The immediate early gene zif268 is a marker for cellular activity and may serve as an important molecule regulating reconsolidation and restabilization of the memory trace^32–35^. Lys48-linked polyubiquitin chain (Ub-Lys48) linkage is known to target proteins for degradation following retrieval, making it a marker for proteasome dependent protein degradation which has been firmly established as a critical regulator of memory destabilization following retrieval^14,17,36^. Thus, we used these markers to compare cells that were active versus cells that had increased protein degradation following retrieval. We performed a procedure identical to our last experiment except that amygdala slices were collected 90 min after standard retrieval or our retrieval-update procedure (Fig. 5a). We then examined the number of zif268 and Ub-Lys48 positive cells in the basolateral amygdala using immunofluroescence (Fig. 5b). We found that vehicle infused groups in the Update condition showed elevated zif268 (*F*_(3,61)_ = 10.83, *P* < 0.001; Fig. 5c) and Ub-Lys48 expression (*F*_(3,61)_ = 10.81, *P* < 0.01; Fig. 5d) in comparison to the Standard retrieval condition, suggesting that our updating procedure enhanced the expression of mechanisms involved in the destabilization and restabilization phases of the reconsolidation process. Furthermore, our retrieval update procedure resulted in a dramatic increase in the number of cells positive for both zif268 and Ub-Lys48 (*F*_(3,57)_ = 2.896, *P* < 0.05); Fig. 5e), suggesting that these mechanisms colocalize in the same cells following retrieval-update procedures. Remarkably, while βlac resulted in increased Ub-Lys48 levels, which is consistent with loss of proteasome function, it did not prevent the update-induced increases in zif268 expression. However, inhibition of GluR2 endocytosis prevented update-dependent increases in both zif268 and Ub-Lys48 single and dual positive cells. Collectively, these results suggest that while contextual fear memory updating is regulated in the amygdala through GluR2 endocytosis and proteasome activity, changes in GluR2 expression are likely upstream of overall increases in protein degradation. Importantly, these results strongly suggest that in the amygdala the original memory was destabilized and updated with the new context-shock association in the same cell populations.

## Discussion

Memory reconsolidation provides an opportunity to include new information into the original memory trace. Reconsolidation-dependent modification of the memory has been known to result in a stronger or weaker fear response and can be used as a therapeutic target to modulate heightened fear responses that are commonly associated with debilitating psychiatric disorders^7,8^. Here, we provide molecular targets for fear memory intervention that does not result in the disruption of the original fear memory. We found that context fear memory retrieval is characterized by changes in trafficking of the GluR2 AMPA receptor subunit in the amygdala, suggesting the exchange of CI-AMPARs for CP-AMPARs. To have a better understanding of how GluR2-containing AMPAR receptor exchange contributes to memory updating, additional shocks were included during memory retrieval which changed fear responses exclusively in groups that had received contextual fear conditioning. Specifically, two low intensity UCS presentations in conditioned groups results in a stronger fear response, while ten low intensity UCS presentations results in a lower fear response. Because the ten UCS update group resulted in less responding, it is unlikely that the increases in fear we see in the two UCS condition are due to shock sensitization or stress enhanced fear learning. Furthermore, the strengthening of the fear response in the two UCS condition was dependent on GluR2 endocytosis and ubiquitin-proteasome system activity, suggesting a critical role for reconsolidation-dependent molecular mechanisms in this memory strengthening effect. The retrieval update effect was characterized by increases in zif268 and Ub-Lys48 in the amygdala, with a significant overlap in zif268 and Ub-Lys48 expressing neurons, which suggests that a number of active cells also undergo destabilization during the retrieval update. Interestingly, inhibition of GluR2 endocytosis prevents elevated cellular activity and ubiquitin tagging as a result of the retrieval update, suggesting that GluR2 trafficking during memory retrieval may regulate ubiquitin-proteasome activity necessary for memory lability and synaptic destabilization. Collectively, these results suggest strengthening of contextual fear memory required GluR2-endocytosis-dependent memory destabilization in amygdala neurons.

Cellular activity and destabilization across an entire neural circuit likely regulates memory strengthening^4,27,37^. Previous studies have highlighted the importance of the dorsal hippocampus, medial prefrontal cortex, and amygdala in memory strengthening dependent on retrieval mechanisms. Our results support previous studies focusing on a critical role for the amygdala in retrieval-dependent fear memory strengthening. Furthermore, previous work has suggested that GluR2 trafficking is necessary for a consolidated memory to become labile following retrieval^13^. The incorporation of GluR2-lacking CP-AMPA receptors and their replacement by GluR2-containing CI-AMPA receptors in principal neurons during LTP is thought to stabilize synapses and aid in memory maintenance and retrieval-dependent updating processes^13,26^. Our results are consistent with these data and demonstrate for the first time that GluR2-containing AMPAR trafficking is critical for memory modification in the amygdala, suggesting memory retrieval is characterized by dynamic changes between CP-AMPA and CI-AMPA receptor trafficking.

Synaptic destabilization during retrieval is necessary to engage memory lability to allow for incorporation of new information into the original memory trace. Ubiquitin proteasome-dependent mechanisms are critical for synaptic destabilization and memory lability in the amygdala^14^. Previous work from our lab shows that systematic elimination of new information during training reduces the ability to disrupt the memory with protein synthesis inhibition during retrieval^38^. Specifically, both context pre-exposure and prediction error during a brief retrieval changed GluR2 surface expression and proteolytic activity in the amygdala, which likely underlie memory lability. Therefore, GluR2 retrieval-dependent trafficking may trigger synaptic destabilization in the presence of new information during a retrieval session to allow for memory updating. Our results are consistent with this work suggesting that GluR2 endocytosis may serve as a trigger for proteasome-dependent protein degradation and the rapid insertion of CP-AMPAR that allows for synaptic restabilization following memory retrieval^13,16^.

While it would be expected that devaluation (i.e, lowering the intensity of) the UCS in the two UCS Update condition would result in a decrease in fear (e.g.^39^) previous work suggests that UCS revaluation is dependent on habituation to the footshock^40^. Since we did not train animals with enough trials to reach asymptote or maximal fear responding to the particular UCS intensity^28^, it is unlikely that groups were habituated to the UCS intensity during training. Based on this, two additional low intensity UCS presentations during a brief retrieval session would then allow for memory modification and a stronger CR while repeated presentations (e.g.^10^) of the low intensity UCS may result in a decreased CR. Other evidence suggests that if the initial learning experience is stressful enough it can result in sensitization of the fear response^30,41^. This could potentially explain our results in the two UCS update condition, as it is possible that the fear response was enhanced by the low intensity shock presentations due to the stressful nature of the initial learning experience^30^. However, this outcome is unlikely given that groups in the ten UCS Update condition show a lower fear response than the Standard retrieval^41^. Therefore, potentiated fear responding from our second day manipulation in the two UCS condition likely occurred because we did not train groups to reach the maximal fear response, which cannot be explained by sensitization of fear or habituation to the UCS for retrieval-dependent revaluation, but rather is likely do to the retrieval session being perceived as additional training^27^.

In summary, these data provide insight about the molecular mechanisms critical for fear memory strengthening following retrieval. We found that the incorporation of new information during retrieval updates the original fear memory, which strengthens fear responses during later tests. Furthermore, the elevated fear responding is dependent on proteasome activity and GluR2 endocytosis, supporting previous work suggesting AMPAR trafficking is critical for destabilization of a fear memory during retrieval. The endocytosis of GluR2 containing AMPARs prevents fear memory strengthening through regulation of the proteasome and reconsolidation specific cellular activity, suggesting that GluR2 endocytosis may trigger destabilization processes necessary for synaptic and behavioral updating during memory reconsolidation. Collectively, these data suggest that targeting of GluR2-endocytosis-dependent reconsolidation destabilization mechanisms may serve as a useful therapeutic strategy for the treatment of stress-induced enhancement of memories for traumatic events.

## Supplementary information

Supplemental figure

## References

[1] McGaugh JL (2000). Memory–a century of consolidation. Science.

[2] McGaugh JL (2015). Consolidating memories. Annu Rev Psychol.

[3] Nader K, Schafe GE, Le Doux JE (2000). Fear memories require protein synthesis in the amygdala for reconsolidation after retrieval. Nature.

[4] Inda MC, Muravieva EV, Alberini CM (2011). Memory retrieval and the passage of time: from reconsolidation and strengthening to extinction. J Neurosci.

[5] Monfils MH, Cowansage KK, Klann E, LeDoux JE (2009). Extinction-reconsolidation boundaries: key to persistent attenuation of fear memories. Science.

[6] Pitman RK (2015). Harnessing Reconsolidation to Treat Mental Disorders. Biol Psychiatry.

[7] Parsons RG, Ressler KJ (2013). Implications of memory modulation for post-traumatic stress and fear disorders. Nat Neurosci.

[8] Alberini CM, Ledoux JE (2013). Memory reconsolidation. Curr Biol.

[9] Soeter M, Kindt M (2015). An Abrupt Transformation of Phobic Behavior After a Post-Retrieval Amnesic Agent. Biol Psychiatry.

[10] Bailey DJ, Kim JJ, Sun W, Thompson RF, Helmstetter FJ (1999). Acquisition of fear conditioning in rats requires the synthesis of mRNA in the amygdala. Behav Neurosci.

[11] Schafe GE, LeDoux JE (2000). Memory consolidation of auditory pavlovian fear conditioning requires protein synthesis and protein kinase A in the amygdala. J Neurosci.

[12] Jobim PF (2012). Inhibition of mTOR by rapamycin in the amygdala or hippocampus impairs formation and reconsolidation of inhibitory avoidance memory. Neurobiol Learn Mem.

[13] Hong I (2013). AMPA receptor exchange underlies transient memory destabilization on retrieval. Proc Natl Acad Sci USA.

[14] Jarome TJ, Werner CT, Kwapis JL, Helmstetter FJ (2011). Activity dependent protein degradation is critical for the formation and stability of fear memory in the amygdala. PLoS One.

[15] Yeh SH, Mao SC, Lin HC, Gean PW (2006). Synaptic expression of glutamate receptor after encoding of fear memory in the rat amygdala. Mol Pharmacol.

[16] Clem RL, Huganir RL (2010). Calcium-permeable AMPA receptor dynamics mediate fear memory erasure. Science.

[17] Lee SH (2008). Synaptic protein degradation underlies destabilization of retrieved fear memory. Science.

[18] Jarome TJ, Helmstetter FJ (2013). The ubiquitin-proteasome system as a critical regulator of synaptic plasticity and long-term memory formation. Neurobiol Learn Mem.

[19] Colledge M (2003). Ubiquitination regulates PSD-95 degradation and AMPA receptor surface expression. Neuron.

[20] Mabb AM (2014). Triad3A regulates synaptic strength by ubiquitination of Arc. Neuron.

[21] Sol Fustinana M, de la Fuente V, Federman N, Freudenthal R, Romano A (2014). Protein degradation by ubiquitin-proteasome system in formation and labilization of contextual conditioning memory. Learn Mem.

[22] Kim J (2007). Amygdala depotentiation and fear extinction. Proc Natl Acad Sci USA.

[23] Kwapis JL, Jarome TJ, Gilmartin MR, Helmstetter FJ (2012). Intra-amygdala infusion of the protein kinase Mzeta inhibitor ZIP disrupts foreground context fear memory. Neurobiol Learn Mem.

[24] Jarome TJ (2012). The timing of multiple retrieval events can alter GluR1 phosphorylation and the requirement for protein synthesis in fear memory reconsolidation. Learn Mem.

[25] Paxinos, G. & Watson, C. *The rat brain in stereotaxic coordinates*. 6th edn, (Academic Press/Elsevier, 2007).

[26] Rao-Ruiz P (2011). Retrieval-specific endocytosis of GluA2-AMPARs underlies adaptive reconsolidation of contextual fear. Nat Neurosci.

[27] Lee JL (2008). Memory reconsolidation mediates the strengthening of memories by additional learning. Nat Neurosci.

[28] Ozawa T (2017). A feedback neural circuit for calibrating aversive memory strength. Nat Neurosci.

[29] Wang SH, de Oliveira Alvares L, Nader K (2009). Cellular and systems mechanisms of memory strength as a constraint on auditory fear reconsolidation. Nat Neurosci.

[30] Poulos AM (2016). Conditioning- and time-dependent increases in context fear and generalization. Learn Mem.

[31] Ghosh S, Chattarji S (2015). Neuronal encoding of the switch from specific to generalized fear. Nat Neurosci.

[32] Hall J, Thomas KL, Everitt BJ (2001). Cellular imaging of zif268 expression in the hippocampus and amygdala during contextual and cued fear memory retrieval: selective activation of hippocampal CA1 neurons during the recall of contextual memories. J Neurosci.

[33] Lee JL, Everitt BJ, Thomas KL (2004). Independent cellular processes for hippocampal memory consolidation and reconsolidation. Science.

[34] Diaz-Mataix L, Ruiz Martinez RC, Schafe GE, LeDoux JE, Doyere V (2013). Detection of a temporal error triggers reconsolidation of amygdala-dependent memories. Curr Biol.

[35] Besnard A, Caboche J, Laroche S (2013). Recall and reconsolidation of contextual fear memory: differential control by ERK and Zif268 expression dosage. PLoS One.

[36] Furini CR (2015). The relationship between protein synthesis and protein degradation in object recognition memory. Behav Brain Res.

[37] Fukushima H (2014). Enhancement of fear memory by retrieval through reconsolidation. Elife.

[38] Jarome TJ, Ferrara NC, Kwapis JL, Helmstetter FJ (2015). Contextual Information Drives the Reconsolidation-Dependent Updating of Retrieved Fear Memories. Neuropsychopharmacology.

[39] Schultz DH, Balderston NL, Geiger JA, Helmstetter FJ (2013). Dissociation between implicit and explicit responses in postconditioning UCS revaluation after fear conditioning in humans. Behav Neurosci.

[40] Davey GC (1989). UCS revaluation and conditioning models of acquired fears. Behav Res Ther.

[41] Poulos AM, Zhuravka I, Long V, Gannam C, Fanselow M (2015). Sensitization of fear learning to mild unconditional stimuli in male and female rats. Behav Neurosci.

