# Supplementary material for: GluR2 endocytosis-dependent protein degradation in the amygdala mediates memory updating
Source: Sci Rep. 2019 Mar 26;9:5180. doi: 10.1038/s41598-019-41526-1 (PMC6435726; doi:10.1038/s41598-019-41526-1)

Supplemental Figure for:

GluR2 endocytosis-dependent protein degradation in the amygdala mediates memory updating

Nicole C. Ferrara*, Timothy J. Jarome*, Patrick K. Cullen, Sabrina A. Orsi, Janine L. Kwapis, Sydney Trask, Shane E. Pullins & Fred J. Helmstetter

GluR2

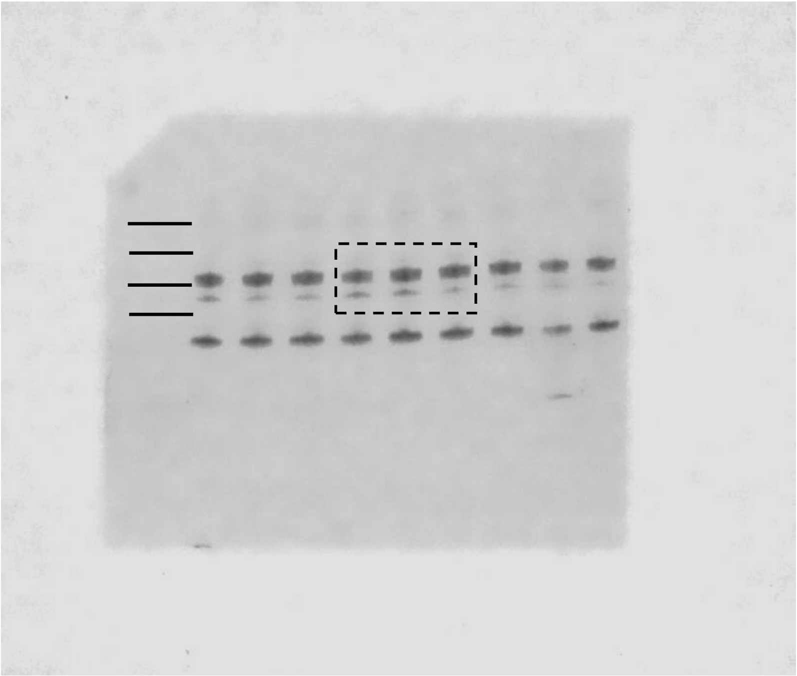

Actin

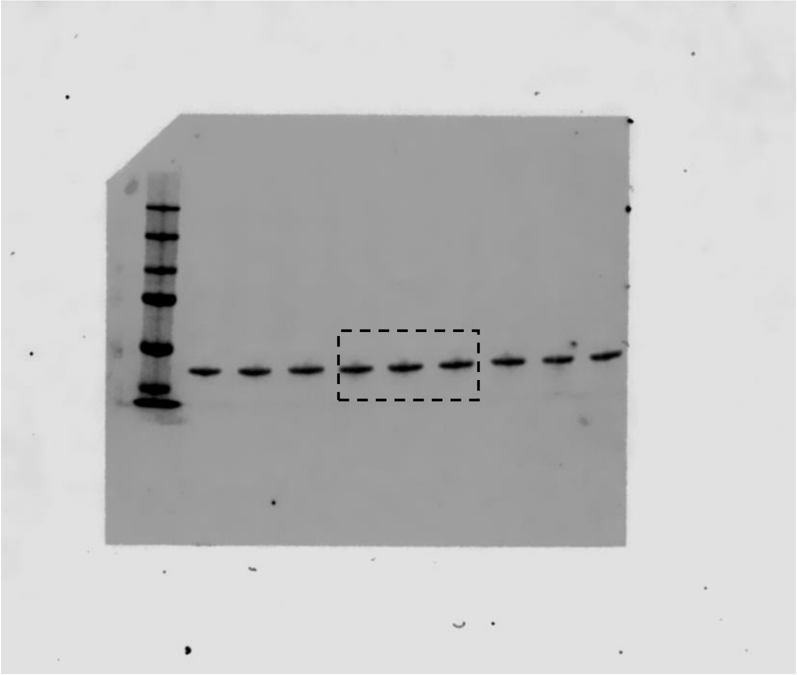

Supplement: Supplementary file 1 — Supplemental figure [file 41598_2019_41526_MOESM1_ESM.pdf]
